# The Paternal Brain in Action: A Review of Human Fathers’ fMRI Brain Responses to Child-Related Stimuli

**DOI:** 10.3390/brainsci11060816

**Published:** 2021-06-20

**Authors:** Livio Provenzi, Johanna Lindstedt, Kris De Coen, Linda Gasparini, Denis Peruzzo, Serena Grumi, Filippo Arrigoni, Sari Ahlqvist-Björkroth

**Affiliations:** 1 Child Psychiatry and Neurology Unit, IRCCS Mondino Foundation, 27100 Pavia, Italy; serena.grumi@mondino.it; 2 Department of Psychology and Speech-Language Pathology, University of Turku, 20500 Turku, Finland; johmat@utu.fi (J.L.); sarahl@utu.fi (S.A.-B.); 3 Neonatal Intensive Care Department, University Hospital of Ghent, 9000 Ghent, Belgium; Kris.DeCoen@ugent.be; 4 Department of Brain and Behavioral Sciences, Università di Pavia, 27100 Pavia, Italy; linda.gasparini01@universitadipavia.it; 5 Neuroimaging Lab, Scientific Institute IRCCS E. Medea, 23842 Bosisio Parini, Italy; denis.peruzzo@lanostrafamiglia.it (D.P.); filippo.arrigoni@lanostrafamiglia.it (F.A.)

**Keywords:** brain, father, fMRI, neuroimaging, parenting

## Abstract

As fathers are increasingly involved in childcare, understanding the neurological underpinnings of fathering has become a key research issue in developmental psychobiology research. This systematic review specifically focused on (1) highlighting methodological issues of paternal brain research using functional magnetic resonance imaging (fMRI) and (2) summarizing findings related to paternal brain responses to auditory and visual infant stimuli. Sixteen papers were included from 157 retrieved records. Sample characteristics (e.g., fathers’ and infant’s age, number of kids, and time spent caregiving), neuroimaging information (e.g., technique, task, stimuli, and processing), and main findings were synthesized by two independent authors. Most of the reviewed works used different stimuli and tasks to test fathers’ responses to child visual and/or auditory stimuli. Pre-processing and first-level analyses were performed with standard pipelines. Greater heterogeneity emerged in second-level analyses. Three main cortical networks (mentalization, embodied simulation, and emotion regulation) and a subcortical network emerged linked with fathers’ responses to infants’ stimuli, but additional areas (e.g., frontal gyrus, posterior cingulate cortex) were also responsive to infants’ visual or auditory stimuli. This review suggests that a distributed and complex brain network may be involved in facilitating fathers’ sensitivity and responses to infant-related stimuli. Nonetheless, specific methodological caveats, the exploratory nature of large parts of the literature to date, and the presence of heterogeneous tasks and measures also demonstrate that systematic improvements in study designs are needed to further advance the field.

## 1. Introduction

During the past two decades, a global change in fatherhood has occurred, at least in Western societies [1]. The change is mostly prominent in the increase in time that modern fathers spend in direct caregiving (e.g., feeding and playing) [2,3,4]. Specific socio-economic levers that boosted this change in fatherhood include the increased rates of women’s employment [5], the economic crisis of the early 21st century [6], and the introduction or improvement of paid paternal leave [7,8]. These factors resulted in a cultural adaptation of the classic masculine identity for men who become fathers [9], suggesting a shift away from “hegemonic masculinity” toward a “caring masculinity” [10]. Of course, there are still wide individual differences in the degree of fathers’ engagement in direct caregiving and in the phenotypes of fathering behavior [11]. Nonetheless, there is also a growing need and interest to understand the impact of a more prominent role for the paternal figure in the family caregiving environment on subsequent child development [12] and the neurobiological underpinnings of fatherhood [13,14,15]. The present study provides a review of functional magnetic resonance imaging (fMRI) studies conducted so far on the neurological correlates of fatherhood and paternal caregiving in humans with a specific focus on brain responses to infant-related stimuli. This systematic review is warranted to highlight promising lines of research, open questions, and implications for healthy and at-risk developmental conditions.

### 1.1. The Key Role of Fathering Behavior

A rapid literature search on Scopus for “fathering” or “paternal caregiving” revealed that research tagged with these keywords has started to become a stable topic in the international scientific community only after 2000, with a minimum of 25 (year 2003) to a maximum of 118 (year 2018) annually published papers. This rapidly accumulating amount of research highlights the importance of fathering and direct fathers’ engagement in daily caregiving for infants’ development, especially during the first months and years of life [16,17]. Gettler and colleagues [18] recently argued that paternal caregiving may be critical in at least three major domains: direct care and teaching, social capital cultivation, and reduction of family conflicts. Meta-analytic evidence suggests that higher paternal sensitivity is associated with child attachment security [19]. The specificity of the paternal role is also highlighted by studies that showed how fathers and mothers engage in different interactive patterns and activities [20] and that higher paternal engagement in direct caregiving may result in a greater heterogeneity of stimuli provided to the child by parents [21]. Paternal behavior has also been shown to promote children’s language and cognitive development [21,22]. The involvement of fathers in active co-parenting has been found to be predictive of more positive socio-emotional development in a sample of 9-month-old infants in at-risk families [23]. Lastly, during a visual cliff paradigm, paternal—but not maternal—expressed anxiety was significantly and positively associated with infant expressed anxiety and task avoidance, suggesting the early establishment of a non-verbal and implicit emotional resonance between fathers and infants at 10–15 months of age [24]. In summary, previous research suggests that fathers may be active contributors to infants’ health by providing sensitive responses to the child, supporting embodied affective mirroring, and promoting emotional regulation and socio-cognitive abilities. Despite the well-documented importance of paternal caregiving for child development, the literature on the neurobiology of fathering is still limited.

### 1.2. Neurobiology of Fathering in Animal Models

Most recently, research on fatherhood began to highlight preliminary evidence of the role played by the paternal brain in male caregiving and co-parenting in animal models. These studies suggest that paternal caregiving behaviors may only partially rely on the same neural circuits and networks linked with maternal caregiving [25]. Research conducted on bi-parental rodent species (e.g., *peromyscus californicus*) reported increased neural activity of the medial preoptic area (MPOA) in males following pup exposure [26] as well as increased latency to approach the pup and reduced time spent caregiving after lesions of the basolateral amygdala [27]. In another bi-parental rodent species, namely the prairie vole, fathers exposed to pups have greater activity in the MPOA, amygdala, lateral septum, and stria terminalis [28]. Above these subcortical regions, other cortical regions have also been hypothesized to be involved in the paternal brain complex network. For example, in non-human primates (i.e., marmoset), vasopressin receptor density on neurons in the prefrontal cortex was higher in fathers than in non-father counterparts [29]. It has recently been hypothesized that cortical networks underpinning paternal caregiving might include brain areas involved in mentalization (e.g., superior temporal sulcus, STS; prefrontal cortex, PFC), embodied simulation (e.g., anterior insula; premotor areas; inferior parietal lobule), and emotion regulation (orbitofrontal cortex, PFC; inferior frontal gyrus, IFG) [25,30]. This hypothetical model presents intriguing overlapping areas with the specific dimensions and roles of paternal caregiving highlighted in the previous paragraph. Consistently, it further suggests that the male brain appears to adapt to the transition to fatherhood to provide the neural underpinnings for effective fathering behavior [17,25].

### 1.3. Challenges in Exploring the Human Paternal Brain

Evidence of the involvement of specific networks in the paternal brain of human fathers are gradually accumulating. For example, a recent meta-analysis performed by Witteman et al. [31] systematized the literature on the neural network involved in infant cry perception, testing as moderator parenthood and gender. The results indicated that males exhibited a larger activation in the right triangular IFG and left angular gyrus compared to females, suggesting that males may activate more semantic processing to evaluate infant cries. Moreover, when parents were compared to non-parents, a higher activation of the bilateral auditory cortex, posterior insula, dorsal anterior insula, pre- and post-central gyrus, and right putamen was observed.

Nonetheless, there is wide variations in the methodology used by previous research in this field, which challenges the drawing of a coherent picture of what pertains to the human paternal brain [15,30]. Heterogeneity in hardware, study design, stimulus choice, and presentation—as well as the different approaches used to analyze the data—represents the first barrier to achieving an integrated comprehension of paternal brain fMRI studies. This is especially true for readers who may be partially familiar with neuroimaging techniques and related methodological issues. Furthermore, even if task stimuli and control conditions are carefully designed to investigate and highlight specific processes of brain activity, it is not possible to completely isolate the brain response to a single stimulus from the surrounding environment, either considering other brain networks or secondary stimulus features. These issues make the interpretation of the results and the comparison among different studies pretty challenging. As such, to provide a comprehensive and rigorous overview of human paternal brain research to date, we included the investigation and the description of previous fMRI studies’ methodology as a key goal of the present review. The appreciation of findings in the context of a careful description and review of methods is warranted to contribute to a better understanding of the knowledge accumulated so far on the human paternal brain and may also benefit future research by highlighting methodological issues that can be improved.

### 1.4. The Present Study

We report on a systematic summary and integration of the evidence to date of paternal brain response to infant-related visual or auditory stimuli. By narrowing the focus of the review to neurological responses to visual or auditory infant-related stimuli, we aimed to provide as close an overall picture as possible to the daily neurological functioning of a father’s brain while interacting with his infant; in other words, an integrated view of the paternal brain *in action*. The primary aim of this review was to investigate which specific brain areas are activated in fathers in response to infant-related stimuli. According to previous research [25], it is plausible to speculate that three major cortical circuits are involved in paternal caregiving and fathering: a mentalizing network, an embodied simulation network, and an emotion regulation network. We also hypothesized a subcortical mammalian parenting network to further support an integrated brain circuitry that underpins paternal behavior. This model was used to guide data abstracting. A secondary aim—yet preliminary to better frame the main goal findings—was to highlight the methodological aspects of paternal brain studies to date. As these methodological issues are key to interpretation of the main findings, they are reported first.

## 2. Materials and Methods

### 2.1. Literature Search

The systematic review was performed according to the Referred Reporting Items for Systematic Review and Meta-Analysis (PRISMA) guidelines [32]. Records were searched on three databases (i.e., PubMed, PsycINFO, and Scopus) to identify eligible studies published until February 2020. The search targeted studies focusing on the paternal brain. The following search string was used: “neuroimaging OR functional magnetic resonance imaging OR magnetic resonance imaging” AND “father OR father–child relationship”. Medical subject headings (MeSH) were used where possible.

### 2.2. Selection of Retrieved Records

**Figure 1:**
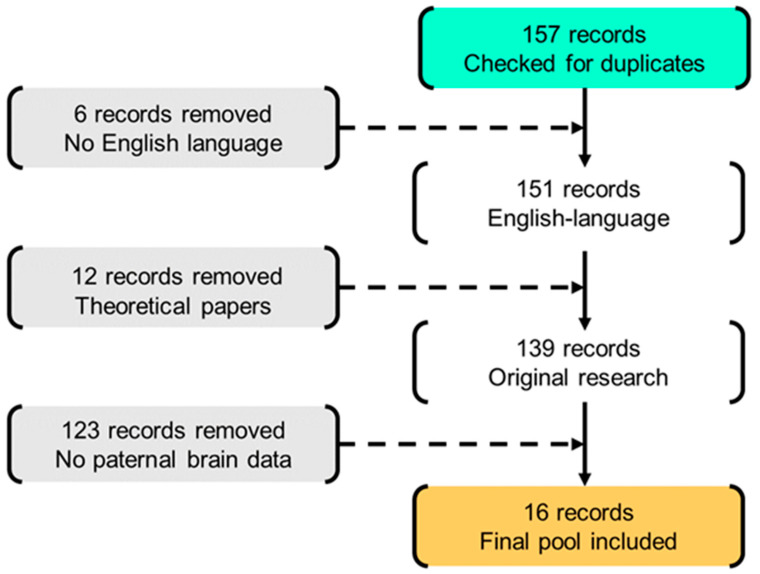
Flow chart of study selection.

The complete flow chart of the study selection is shown in Figure 1. Cross-referencing did not reveal any additional studies to be included. The retrieved records were first filtered independently by two authors (LP and JL) for potentially relevant articles by screening titles, abstracts, and the full articles. Disagreement was solved in conference. Duplicate records were first identified and removed. Only fMRI studies were included. Moreover, only studies that included fathers of children up to preschool age were selected to obtain a more homogeneous sample focused on the early stages of development. Only peer-reviewed, English language papers, reporting original results were included. No restrictions were set for fathers and/or infants’ clinical conditions, publication time, or type of experimental task.

### 2.3. Data Abstracting

The following data were abstracted: general information (authors, year of publication, title, journal), sample characteristics (sample size, fathers’ age, infants’ age, number of father offspring, marital status, fathers’ participation in caregiving), methodology (use of control groups, type of stimulus, study design), neuroimaging acquisition (sequence set up) and processing (analysis technique), and findings (exploratory results, region-of-interest results).

### 2.4. Data Synthesis

The data were synthesized according to the aims of the present review. Study characteristics (sample and study design details) were reviewed first; this included a detailed report on sample size and fathers and infants’ variables, as well as on the methodological design of original studies. The primary findings of the original studies were attributed to four different networks: mentalizing network, embodied simulation network, emotion regulation network, and subcortical mammalian parenting network. All findings reporting brain areas or circuits that did not fall into these three major networks previously identified in animal model research on paternal caregiving were synthesized into a fifth category (i.e., other brain regions). The retrieved synthesis was shared among the authors of this review for discussion and refinement.

## 3. Results

### 3.1. Participant Characteristics

**Figure 2:**
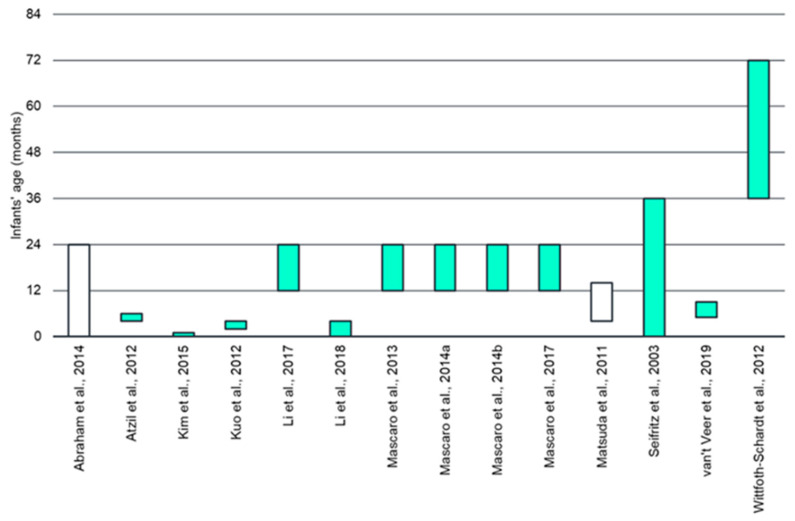
Offspring age range for the included studies [34,35,36,37,38,39,40,41,42,43,44,45,46,47]. Note. Two studies were not included because the data were not available (De Pisapia et al., 2013) [33] or were related to unborn infants (Thijssen et al., 2018) [48]. Please, note that in Abraham et al. (2014) [44] and Matsuda et al. (2011) [47] the minimum and maximum ages were not available. Nonetheless, mean and standard deviations were reported, and an estimated range was obtained by applying ± two standard deviations to mean age.

Sixteen studies were included in the systematic review (Table 1). The sample size varied widely among the included studies, ranging from five [33] to 88 [34]. Nine papers reported the number of children for the enrolled fathers, which ranged from zero (i.e., expecting fathers) to a maximum of five. All fathers were cohabitants with the mothers of their infant(s). The time spent caregiving was not systematically detailed; only one study [35] reported descriptive statistics for this variable (i.e., 33 h per week on average). Infant’s age ranged from zero to 72 months (see Figure 2). All fathers and infants were healthy.

### 3.2. Methodology Review

The tasks involved the exposure of fathers to visual or auditory stimuli of their own infant or another familiar or unknown infant while undergoing an fMRI scan. Six studies used only stimuli of an unknown infant, while ten studies also included fathers’ own infant stimuli. An overview of the stimuli used is reported in Table 2.

In total, 18 different stimuli were investigated in 16 manuscripts, as a two-task combination was included in two of the selected records. Most of the studies investigated infant audio stimuli (crying, laughing, vocalization, speech) (eight tasks) or infant face pictures (five tasks). The remaining stimuli included pictures or videos of father–infant interactions. Nine out of the 18 tasks directly investigated the contrast of their own vs. an unknown infant (e.g., their own infant face vs. an unknown infant face), while the others modulated different aspects of the stimulus, such as its intrinsic emotional content (e.g., crying vs. laughing). Ten of the 16 studies focused only on fathers, while three studies compared fathers to non-father males, five compared fathers to mothers, and one included mixed groups (male/female, parent/non-parent) to investigate gender- and parenthood-related differences.

Fifteen tasks were administered according to a block design paradigm, while only three used an event-related paradigm. The fMRI sequence parameters are reported in Table 3. Most studies were performed on a 3 T scanner (12 out of 16 studies), with the most frequent set up using a 2000 ms or a little longer repetition time (TR), a 30 ms echo time (TE) and a final voxel resolution of 3.5 × 3.5 × 3.55 mm^3^. Only one study [36] applied a TR smaller than 2000 ms, with the drawback of a poor voxel resolution (i.e., 4 × 4 × 4 mm^3^). Almost all studies (14 out of 16) acquired anisotropic voxels, that is with a slice thickness larger than the in-plane resolution, and most of them set a non-zero gap between consecutive slices (9 out of 16 studies). Two studies were performed on 1.5 T scanners [35,37], with a worse combination of TR-voxel resolution than the studies performed on more modern scanners.

All studies included in this review adopted a similar approach to the data analysis. They performed comparable preprocessing and first-level analyses (i.e., in each subject), although they used different approaches in the second-level one (i.e., population level). A “consensus preprocessing pipeline” can be identified including all pre-formed steps in at least 80% of the studies (i.e., at least 13 out of 16 studies); it includes motion correction, slice timing correction, temporal filtering, registration to a common template (e.g., Talairach, MNI), and spatial smoothing. Differences among the studies in the “consensus preprocessing pipeline” essentially concern the execution order of the different steps, the specific parameters set up (e.g., the smoothing level, the common template space, etc.,), and the software used to perform the analysis. Further preprocessing steps include brain extraction (4 out of 16 studies), outlier volume detection either for removing the whole subject or for removing outlier volumes (5 out of 16 studies), and use of nuisance regressors (e.g., motion regressors, instruction stimuli, patient response) in the first-level analysis (5 out of 16 studies). After the preprocessing, all studies used a linear model to identify the activation patterns for each subject related to the different experimental conditions that were used in the second level analysis. In the second level analysis, all studies first performed a voxel analysis using linear models, at least for exploratory purpose [38,39].

However, differences emerged in the linear model selection (e.g., random effects, mixed effects), in the selection of the clinical covariates, and in the selection of the statistical test to be performed on top of the linear model. Almost all studies (13 out of 16) also exploited some regions of interest (ROIs) to better focus their analyses. ROIs were selected either a priori (five studies), data-driven (six studies), or defined with a mixed approach (two studies). A priori ROIs were selected on the basis of physiological hypotheses or from previous studies. Data-driven ROIs were usually obtained by performing a preliminary second-level analysis to identify the brain regions associated with the specific stimulus implemented in the study, thus using the same data that will be used in the subsequent statistical analyses. In the mixed approach, the a priori selected ROIs were further refined using a population level analysis. ROIs were exploited in different ways. In the most common approach (10 studies), the average activation value for each subject in the given ROIs was computed, thus improving its signal to noise ratio and statistical power, and used as input for further statistical analyses (e.g., ANOVA, correlation, etc.,). Differently, three studies used the selected ROIs as a mask to restrict the voxel statistical analysis, thus avoiding spurious activations/statistical differences and reducing the amount of type I errors. Table 4 reports a summary of the different methodological choices performed in all the studies.

### 3.3. Emerging Paternal Brain Areas

#### 3.3.1. Mentalizing Network

Two main areas related to mentalization have been reported as significantly associated with human fathers’ exposure to their own infant stimuli: the superior temporal sulcus (STS) [40,49] and the medial prefrontal cortex (MPFC) [33,41,42,43,49]. Higher STS activations were reported when fathers were exposed to videotaped interactions of themselves and their own 11-month-old infants compared to similar videos of unfamiliar father–infant pairs [49]. Similar findings were obtained for static pictures of their own 1–3-year-old children compared to pictures of unfamiliar children or adults [40]. The MPFC involvement was reported in response to videotaped interactions [49] and static pictures of the own 1–2-year-old children compared to an unknown child and to an adult stimulus [42]. In another study, the activation of both STS and MPFC emerged in response to cry acoustics of both own and unfamiliar children during the first four months of life [43].

#### 3.3.2. Embodied Simulation Network

The insula was reported to be more active in fathers exposed to videotaped interactions of themselves and their infants compared to unknown infants’ stimuli [41,49]. Anterior insula (AI) was more active in men when looking at emotional infant pictures compared to adult ones [38]. Other circuits known to be part of the embodied simulation network have also been reported by single studies. Mascaro and collaborators [38] showed higher activation of the middle and lateral superior frontal gyrus (SFG) in response to 1–3-year-old infants’ pictures in fathers compared to non-father counterparts. The ventral anterior cingulate cortex (vACC) activation was reported by Abraham and colleagues [44] in fathers looking at previously videotaped interactions with their own infants at the end of the first year of life. The juxtapositional lobule emerged as significantly more active in fathers watching video clips of their own infants in potentially threatening settings, compared to unknown infants in similar contexts [39].

#### 3.3.3. Emotion Regulation Network

The inferior frontal gyrus (IFG) has been reported in multiple studies in response to a different set of infants’ stimuli (crying) [43], static pictures [40], and brief videotapes [36]. Li and colleagues [43] reported similar activation of this area in response to their own and to a stranger infant crying sound, whereas Kuo and colleagues [36] highlighted a more pronounced IFG activation in response to videos of their own infant compared to stranger ones. Mascaro and collaborators [38] reported a higher IFG response in fathers exposed to unfamiliar infant vs. adult face stimuli. Looking at infant pictures—compared to adults’ ones—was significantly associated with an increased activity of the orbitofrontal cortex (OFC) in men [34]. This activation was higher in fathers compared to non-father counterparts. Additionally, fathers of 2–4-months-old infants also exhibited greater OFC activation while watching previously videotaped 15-sec interactions with their own infants compared to unknown adult–infant pairs [36]. The same area was more active in response to a positive emotionality face picture of the own infant in fathers of 12–24-month-old females compared to fathers of age-matched males [45].

#### 3.3.4. Subcortical Parenting Network

Many subcortical structures emerged as significantly activated when fathers were exposed to infants’ stimuli. These included the caudate [35,36,43,46], the putamen [42], the pallidum [35], the globus pallidus [38], the thalamus [42,43,46], the substantia nigra [42], and the amygdala [35,37,44]. In Wittfoth-Schardt’s paper [35] the own infant stimulus was linked with increased left pallidum response compared to a familiar infant stimulus and with increased right pallidum and left amygdala’s ventral tegmental area compared to an unfamiliar infant stimulus. Notably, Li and colleagues [43] did not find any significant difference in the neural response of first-time fathers to the crying sound of unknown and own 4-month infants. Nonetheless, the same authors reported increased activation of the thalamus to the own infants’ stimuli compared to unknown adult ones [42].

#### 3.3.5. Other Brain Regions

Other brain regions emerged as being associated with paternal neural responses to infant stimuli. Both the superior frontal gyrus (SFG) [36] and the precentral gyrus (PrG) [40] were more responsive to the own infant, whereas the fusiform gyrus (FuG) was more responsive to visual infant stimuli vs. adult ones [34] and to unfamiliar infant stimuli compared to own infant videos [36]. Increased activation for own (vs. unknown) infants in threatening (vs neutral) situations emerged in bilateral motor areas (e.g., parietal operculum, posterior cingulate cortex, lingual gyrus, occipital pole, lateral occipital cortex, and juxtapositional lobule cortex) [33,39,48]. These effects were confirmed both prenatally and postnatally, despite the significant difference between own and unknown infant’s stimuli being no longer significant postnatally.

## 4. Discussion

The present review aimed to obtain an integrated summary of previous studies that investigated the human paternal brain response to child-related auditory and visual stimuli. Preliminarily to the interpretation of the findings, a fine-tuned and in-depth methodological screening of the studies was done to highlight potential biases and caveats as well as to disentangle the variability in task design and neuroimaging data among the included records.

### 4.1. Preliminary Methodological Issues

Considering hardware resources, only two studies used 1.5 T scanners, while the majority were performed on 3 T or 4 T scanners with acceptable TR-voxel resolution. To date, no studies have exploited the advantage of recent multi-band technology [50] that improves the temporal resolution of fMRI data by dramatically decreasing TRs. This is noteworthy, as the temporal resolution is one of the main limitations of the majority of functional studies [51]. Regarding stimuli and tasks, despite a relevant heterogeneity in the choice of stimuli (i.e., audio, images, videos) and modality of presentation (e.g., duration, interstimulus, baseline, control conditions, etc.,), most of the studies investigated two main aspects of fatherhood: the response to different emotional displays and the effect of being exposed to one’s own vs. an unknown child. Noteworthy, all but three studies adopted a block design. An event-related approach was used in only three studies [35,37,47]. Block-design tasks have the advantage of being very simple, yet robust and they require straightforward analysis. Nonetheless, they are more prone and susceptible to subject habituation and they have lower temporal resolution compared to event-related tasks [52].

Pre-processing of fMRI data was performed by using standard steps for artifact detection and correction and for spatial normalization/alignment as well as canonical general linear modeling that was applied to model the BOLD signal. Large differences emerged among the included studies in the second level analysis and in the use of specific ROIs. This is not surprising in fMRI studies, as the population-level statistical analyses are commonly customized to the specific hypotheses to test. In most of the selected studies, a whole-brain analysis did not highlight significant results, often due—at least partially—to the correction needed to account for type I error when performing multiple comparisons (one for each brain voxel) with the same statistical analysis on correlated data (close voxels are strongly correlated). Some studies showed the results obtained using uncorrected *p*-values (i.e., not performing the correction for multiple comparisons), but the reliability and the reproducibility of these results are weak. The use of specific ROIs can overcome this limitation, both by reducing the number of experiments to correct for and by improving the SNR of the data. In the studies reviewed here, ROIs selection was made “a priori,” on the basis of previous results—usually from animal model research. In some cases it was built on biological-behavioral association hypotheses. In fewer studies, the choice of ROIs was data-driven—usually based on whole-group whole-brain analyses.

Both methods (theory- and data-driven) are commonly accepted; notwithstanding, limitations should also be acknowledged. The “a priori” ROI selection is suitable for verifying a single hypothesis (e.g., the involvement of a brain area in a specific cognitive/emotional process); however, it prevents the identification of unexpected, additional circuitry that may cooperate in the task [53,54]. Data-driven approaches allow to identify more brain areas that are potentially involved in the task; nonetheless, when both the ROI selection and the subsequent statistical analyses are performed on the same (small) sample, the results may become self-referential and biased. A good practice should be to perform and show the results from a whole-brain analysis in ROI-based studies. This may further support the rationale for ROIs selection, strengthen ROI-related results, and help formulate new hypotheses.

### 4.2. Toward an Integrated View of the Human Paternal Brain

**Figure 3:**
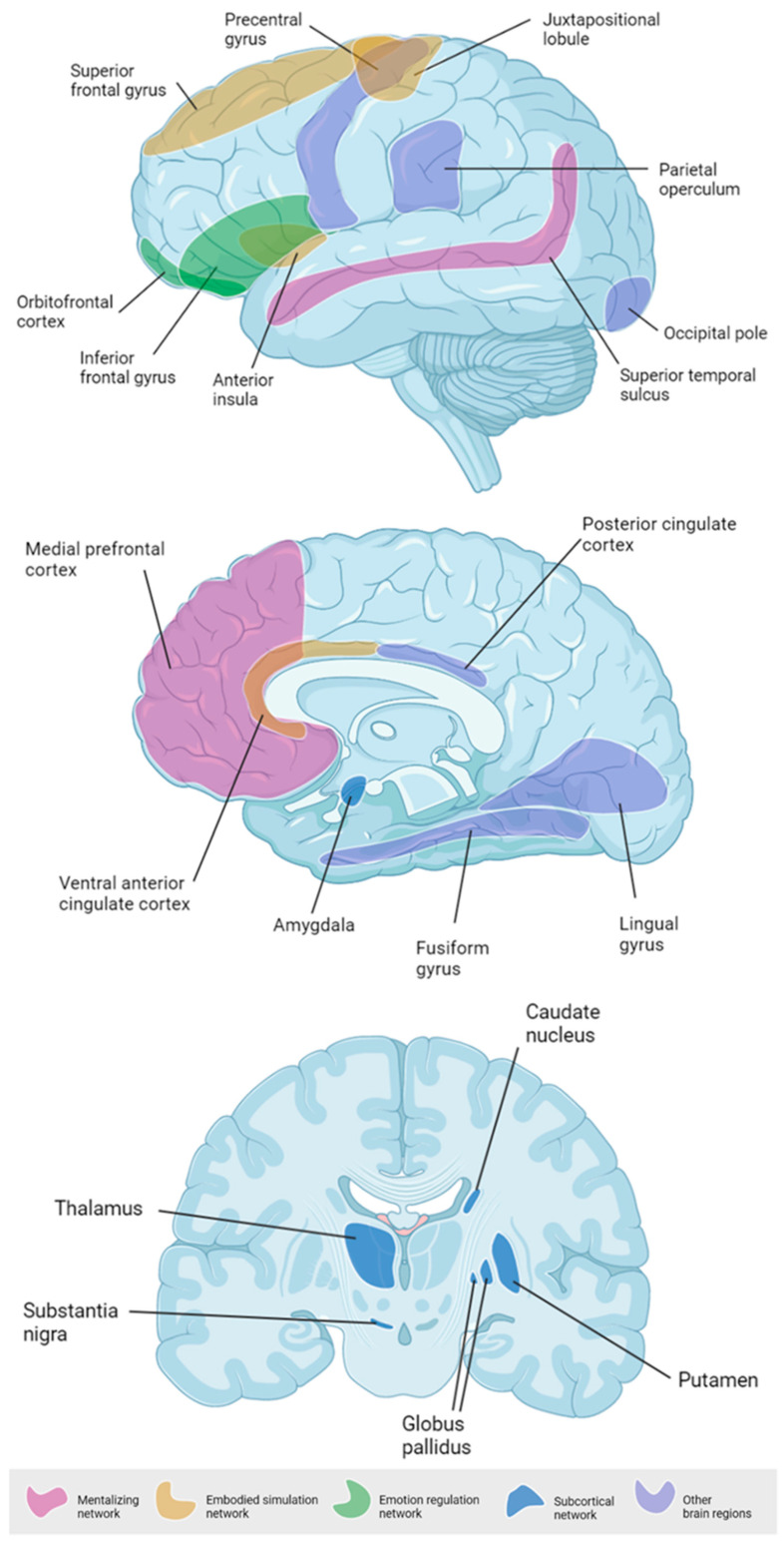
Schematic representation of brain regions activated by the exposure to child-related stimuli in fathers.

Our review largely supports the involvement of three major cortical brain networks that can be ascribed to the so-called “paternal brain” model (Figure 3) [25]. The role of these networks adds to our comprehension of fathering. First, mentalization-related brain areas such as the STS have been linked with the neural processing of human faces and voices [55,56], social perception [57], and the understanding of others’ actions, intentions, and mental states [58,59]. Similarly, the MPFC is involved in the processing of others’ emotional states, regardless of the type of stimulus (e.g., visual or audio) [60,61]. The recruitment of these areas while interacting with their own infant or being exposed to related stimuli is not surprising, as fathers have to pay attention to social cues that facilitate the understanding of infants’ mental and socio-emotional status. Previous research has highlighted that fathers have similar capacity to understand and interpret infants’ social cues and to produce mind-related comments when compared to mothers [62]. The recruitment of these circuits may sustain the observed capacity of fathers to provide accurate interpretations of their infants’ social cues. 

Second, the embodied simulation network appears to be specifically activated in the paternal brain while watching or hearing their own infants’ stimuli. This network is thought to facilitate an immediate grasping of others’ intentions and to favor an emotional resonance between two interacting individuals [63]. Among the regions included in this network, a significant increase in the activation of the anterior insula in the fathers’ brain was reported by different studies in response to both visual [44] and auditory [42] stimuli of their own infants. The anterior insula is a key brain area involved in the integration of interoceptive signals into a person’s body image and in the integration of exteroceptive signals into a cohesive sense of self [64,65,66]. Notably, anterior insula develops early during fetal life and reaches an advanced maturational stage at 27 weeks of gestation [67]. Recent research suggests that the insula may be involved in the early development of self–other rudimentary differentiation in infants [68] and early bodily interactions with parents appear to play a pivotal role in promoting such milestone development during the first months of life [66]. Nonetheless, while previous studies on the role of bodily interactions with the caregiver mainly focused on the mother [69,70,71], very little is known about the role played by fathers’ physical stimulations. Previous research suggests that mothers and fathers may engage in different patterns of social touch with their infant [72,73] and that fathers may mirror and resonate specific affective expressions during social-cognition tasks [74]. Future studies are warranted to explore how the anterior insula sensitivity to the own infants’ stimuli may support such an embodied promotion of infants’ early socio-cognitive development.

A third brain network—that is the emotional regulation network—was also reported. These include the IFG [75] and the OFC [76]. The IFG is activated in response to the perception of emotional displays [77], and it appears to play a pivotal role in emotion appraisal [77,78], a process that is key to parental caregiving and sensitivity [79,80]. The OFC activation is thought to enhance amygdala and hippocampus activity, two subcortical regions involved in emotion regulation and memory formation [81]. From this perspective, it is intriguing to imagine that the integrated activation of the emotional regulation network and subcortical mammalian brain areas may support the creation of infant-related memories that may ultimately promote appropriate sensitive and reliable caregiving behaviors in the father. Notably, previous research on mothers highlighted that caregiving sensitivity is linked to regions involved in the same networks activated in the paternal brain, such as the anterior insula, IFG, hippocampus, and MPFC [82]. It is plausible to hypothesize that paternal sensitivity may also build on the same brain regions involved in maternal sensitivity, further supporting the notion that it is the direct involvement in primary caregiving that shapes a parent’s brain partially regardless of parents’ sex.

Other brain regions not previously included in the paternal brain model emerged from this review. These include bilateral motor areas that can add to the embodied simulation network [83,84]. For example, the juxtapositional lobule (formerly known as the supplementary motor cortex) is involved in the creation of time-dependent motor memories [85] which are key in developing expectancies in self–other interactions such as parent–infant exchanges [86]. The SFG was previously linked to the brain response of mothers to the crying sound of their own infants and the activation of these areas was increased in breastfeeding mothers [87]. It may be plausible that the same area may be involved in the paternal brain response, even if Kuo and colleagues [36] used visual stimuli and the specific involvement of the SFG needs further explorations. The PrG is involved in the execution of voluntary movements and it is plausible to speculate that it may be recruited by the paternal brain to prompt appropriate fathering behaviors in response to infants’ stimuli, as previously hypothesized for mothers [88]. Finally, larger FuG volume has been documented in women reporting higher quality of care during their own childhood [89] which may suggest that this area may be involved in the intergenerational transmission of sensitive parenting across generations.

### 4.3. Limitations

One of the most important points raised by this systematic review is that the paternal brain is highly responsive to infant stimuli. Nonetheless, the available literature surprisingly lacks enough details on the actual time spent by fathers in direct caregiving activities during daily life. This is a major limitation of studies so far, as it dramatically reduces the possibility of making further hypotheses on the effective role played by direct experience in fathering in linking transition to fatherhood and functional changes in brain reactivity to infant stimuli. From a methodological point of view, there is substantial variability among the included studies in terms of sample size (i.e., 5 to 88), with half of the studies involving fewer than 25 participants. This may imply potential limitations in the statistical power of the original analyses. Moreover, it should be highlighted that a recent meta-analysis failed to witness the test-retest reliability of task-functional MRI [90]. These methodological issues, together with the limited number of studies, did not allow us to perform a meta-analytical synthesis and limited the generalizability of the present results. It should also be noted that within the selected literature there was substantial overlap of papers produced by the same lab. Two papers [41,44] are from Ruth Feldman’s lab in Israel, two papers are published by the group of van IJzendoorn [39,48], and six papers are from Mascaro’s research team [34,38,40,42,43,45]. Only five papers out of sixteen (31%) are produced independently by different labs. Although this issue is obviously related to the availability of fMRI resources in a limited number of parenting-focused lab facilities, it should be highlighted that the findings reported in the present systematic review are highly interdependent across publications. This issue calls attention to the need for additional research from multiple research groups and/or on multiple samples of fathers to generate replicable findings and eventually increase generalizability. Furthermore, the research reviewed here provided information on fathers of infants aged 0–2 years (with the exception of Seifritz et al. [37] (0–3 years) and Wittfoth-Schardt et al. [35] (3–6 years)). Nonetheless, the actual minimum and maximum age of infants were not reported in two studies [44,47]. Despite the fact that brain areas do not seem to cluster by infants’ age in the reviewed literature, the variability in task design and infant stimuli did not allow to conduct a proper meta-regression on the relationship between infants’ age and paternal brain response. Nonetheless, this remains a relevant goal of future longitudinal studies. As recent research suggests that the quality of paternal interactive behavior and dyadic interaction may increase from 4 to 18 months of infants’ age [91], acquiring information on the timing of paternal brain adaptation to fatherhood appears key to identifying potential developmentally sensitive windows. Finally, regarding data analysis, no studies included resting-state fMRI or performed connectivity analysis on the neural circuitry of fathers based on fMRI tasks. The analysis of functional connectivity can reveal interactions and interdependence of brain areas, thus helping to better frame the development and characteristics of complex neural networks related to fatherhood. This kind of approach is highly encouraged as it could provide evidence supporting the models of brain interactions that emerged from traditional task-based fMRI studies.

## 5. Conclusions

The present systematic review suggests that child-related visual and auditory stimuli activate a paternal brain network that includes, but is not limited to, three interconnected subnetworks that regulate embodied simulation, mentalization, and emotion regulation and recruit subcortical areas that have relevant roles in the socio-emotional elaboration of child-related stimuli. However, this conclusion should be considered with caution, as several shortcomings of the studies to date (e.g., underpowered sample size) have been highlighted, and different brain regions may serve multiple functions. As previous research on mothers showed that caregiving sensitivity is underpinned by regions involved in the same networks activated in the paternal brain [82], it is plausible to hypothesize that paternal caregiving sensitivity maybe built on these networks. Although previous research and reviews have focused on the hormonal and brain underpinnings of mammalian paternal caregiving [92,93], the present systematic review further adds to the research field by providing an updated, human-focused review that specifically reports on fathers’ functional brain responses to child-related stimuli. It should be highlighted that the study of live brain-to-brain interactions is increasingly becoming possible due to hyper-scanning techniques [94,95,96]. Consistently, the present systematic presentation of paternal neural networks that may be recruited in live father–child interactions appears to be timely and it might benefit future studies in this promising neuroscientific and developmental field.

Future research is warranted to test how the complex paternal network is associated with fathers’ caregiving sensitivity and, in a broader sense, the quality of fathers’ caregiving behavior. Overall, the review provides a partial understanding of the activation of fathers’ brain networks, as it does not take into account the amount of father involvement in direct caregiving, the developing nature of father–child relationship, and other sensory stimuli characteristic of the father–child relationship. The focus on functional connectivity analysis could unveil the development and characteristics of complex neural networks related to fatherhood. The use of advantageous study designs, such as longitudinal studies starting from transition to fatherhood and including emotional and physical dimensions of paternal closeness, would also generate a deeper understanding of human fatherhood, useful for research advancement and clinical purposes.

## Figures and Tables

**Table 1:** Schematic description of the core characteristics of the included studies.

Study	Journal	Fathers N	Other Group(s)	Stimulus Type	Brain NetWork(s) ^§^
Abraham et al., 2014 [44]	PNAS	21	Mothers; homosexual fathers	Visual	M, ES
Atzil et al., 2012 [41]	JAACAP	15	Mothers	Visual	M, ES
De Pisapia et al., 2013 [33]	Neuroreport	5	Mothers; non-parent adults	Sound	M, O
Kim et al., 2015 [46]	Front Psychol	19	Mothers	Sound	SC
Kuo et al., 2012 [36]	Biol Psychol	10	N/A	Visual	ER, SC, O
Li et al., 2017 [42]	Horm Behav	31	N/A	Sound	M, SC
Li et al., 2018 [43]	Physiol Behav	42	N/A	Sound	M, ER
Mascaro et al., 2013 [40]	PNAS	70	N/A	Visual	M,
Mascaro et al., 2014a [38]	SCAN	36	N/A	Sound	ES, ER, SC
Mascaro et al., 2014b [34]	PNEC	88	Non-father men	Visual	ES, ER, O
Mascaro et al., 2017 [45]	Behav Neurosci	69	N/A	Visual	ER
Matsuda et al., 2011 [47]	Neuroimage	15	Mothers; non-parent men	Sound	NSA
Seifritz et al., 2003 [37]	Biol Psychiatry	10	Mothers; non-parent women	Sound	SC
Thijssen et al., 2018 [48]	Horm Behav	25	N/A	Sound	O
van ‘t Veer et al., 2019 [39]	SCAN	21	N/A	Visual	ES, O
Wittfoth-Schardt et al., 2012 [35]	Neuropsychoph	19	N/A	Visual	SC

^§^ Based on Feldman et al. 2019. N/A, not applicable; M, mentalizing; ES, embodied simulation; ER, emotion regulation; SC, subcortical regions; O, others; NSA, no significant associations.

**Table 2:** Overview of the stimuli used in paternal brain research.

Stimulus Condition	Tasks	References
Auditory stimuli		
Crying/laughing	6	De Pisapia et al., 2013 [33]; Kim et al., 2015 [46]; Li et al., 2017 [42]; Mascaro et al., 2014a [38]; Seifritz et al., 2003 [37]; Thijssen et al., 2018 [48]
Infant-directed speech	1	Matsuda et al., 2011 [47]
Baby vocalization	1	Mascaro et al., 2014a [38]
Visual stimuli		
Face pictures	5	Li et al., 2017 [42]; Mascaro et al., 2014b [34]; Mascaro et al., 2013 [40]; Wittfoth-Schardt et al., 2012 [35]; Mascaro et al., 2017 [45]
Father-infant videotapes	3	Abraham et al., 2015 [44]; Atzil et al., 2012 [41]; Kuo et al., 2012 [36]
Infant alone videotapes	2	Atzil et al., 2012 [41]; van’t Veer et al., 2019 [39]

**Table 3:** Summary of the most common fMRI sequence parameters.

Study	Field Strength	TR [ms]	TE [ms]	Slice Thickness (Slice Gap) [mm]	In Plane Resolution [mm^2^]	Number of Slices
Abraham et al., 2014 [44]	3 T	3000	35	3	2.33 × 2.33	39
Atzil et al., 2012 [41]	3 T	3000	35	3	1.56 × 1.56	39
De Pisapia et al., 2013 [33]	4 T	2000	33	3 (0.45)	3 × 3	34
Kim et al., 2015 [46]	3 T	2000	30	5	3.125 × 3.125	30
Kuo et al., 2012 [36]	3 T	1500	30	4	4 × 4	28
Li et al., 2017 [42]	3 T	2000	28	2.5 (1.05)	3.5 × 3.5	34
Li et al., 2018 [43]	3 T	2380	30	3	3 × 3	38
Mascaro et al., 2013 [40]	3 T	2000	28	2.5 (1.05)	3.5 × 3.5	34
Mascaro et al., 2014a [38]	3 T	2000	28	2.5 (1.05)	3.5 × 3.5	34
Mascaro et al., 2014b [34]	3 T	2000	28	2.5 (1.05)	3.5 × 3.5	34
Mascaro et al., 2017 [45]	3 T	2000	28	2.5 (1.05)	3.5 × 3.5	34
Matsuda et al., 2011 [47]	4 T	2600	25	5	3.75 × 3.75	23
Seifritz et al., 2003 [37]	1.5 T	2675	/	4	2.8 × 2.8	25
Thijssen et al., 2018 [48]	3 T	2200	30	2.75 (0.275)	2.75 × 2.75	38
van ‘t Veer et al., 2019 [39]	3 T	2200	30	2.75 (0.275)	2.75 × 2.75	38
Wittfoth-Schardt et al., 2012 [35]	1.5 T	2100	30	3 (0.75)	3 × 3	35

TR, repetition time; TE, echo time.

**Table 4:** Summary of the fMRI analytical methods.

Study	Voxel Level	ROI Purpose	ROI Selection	Summary of Selected ROIs	2nd Level Analysis	Software
Abraham et al., 2014 [44]	Yes	Sampling ROI	Mixed	Amyg, STS, TP, vmPFC, FP	VOXEL: random effect models; ROI: correlations	Brain Voyager
Atzil et al., 2012 [41]	Yes	-	-	-	Random effect models; correlation	Not specified
De Pisapia et al., 2013 [33]	Yes	Inclusive Mask	Data-driven	STL, dmPFC, PCC	Random effect models	Brain Voyager
Kim et al., 2015 [46]	Yes	Inclusive Mask	Theory-driven	Limbic system, striatum, midbrain regions (STG, Put, SN)	Random effect models; Mediation models	SPM8
Kuo et al., 2012 [36]	Yes	Sampling ROI	Mixed	OFC, SFG, mFG, IFG, Cau, SPL, mTL, CG	Random effect models; correlations	SPM8
Li et al., 2017 [42]	Yes	Sampling ROI	Theory-driven	Cau, ACC	Random effect models	FSL
Li et al., 2018 [43]	Yes	Sampling ROI	Data-driven	15 ROIs for different analyses	Mixed effect models; correlation analysis	FSL
Mascaro et al., 2013 [40]	Yes (exploratory)	Sampling ROI	Theory-driven	VTA, SN	VOXEL/ROI: Correlation analysis	Brain Voyager
Mascaro et al., 2014a [38]	Yes	Sampling ROI	Theory-driven	AI	VOXEL: Random effect models, correlation analysis; ROI: correlation analysis	Brain voyager
Mascaro et al., 2014b [34]	Yes	Sampling ROI	Data-driven	mFG, AI, vmPFC, mOFC	VOXEL: random effect model, correlation analysis. ROIs: *t*-test, correlation analysis.	Brain Voyager
Mascaro et al., 2014b [34]	Yes	Sampling ROI	Data-driven	Hyp, Accu, Cau, SN/VTA	VOXEL: random effect model, correlation analysis. ROIs: *t*-test, correlation analysis.	Brain Voyager
Mascaro et al., 2017 [45]	Yes	-	-	-	ANCOVA; correlation analysis	Brain Voyager
Matsuda et al., 2011 [47]	Yes	Sampling ROI	Data-driven	pIFG, SPL, ACC	VOXEL: Random effect model; ROIs: ANOVA, correlation analysis	Brain voyager
Seifritz et al., 2003 [37]	Yes	-	-	-	VOXEL: *t*-test; ROIs:	Brain voyager
Thijssen et al., 2018 [48]	Yes	Inclusive Mask	Data-driven	Infant cry network	2 × 2 models and post-hoc *t*-test, F-test	FSL
van ‘t Veer et al., 2019 [39]	Yes (exploratory)	Sampling ROI	Theory-driven	Amyg	VOXEL: mixed effect model. ROI: GLM repeated measure model (SPSS)	FSL + SPSS
Wittfoth-Schardt et al., 2012 [35]	Yes	Sampling ROI	Theory-driven	GP, Hip	VOXEL: random effect model; ROIs: ANOVA, connectivity analysis	SPM8

ROI, region of interest; ACC, anterior cingulate cortex; Accu, accumbens; AI, anterior insula; Amyg, amygdala; Cau, caudate nucleus; CG, cingulate gyrus; dmPFC, dorsomedial prefrontal cortex; FP, frontal pole; GP, globus pallidus; Hyp, hypothalamus; IFG, inferior frontal gyrus; mFG, middle frontal gyrus; mTL, mediotemporal lobe; OFC, orbitofrontal cortex; PCC, posterior cingulate cortex; pIFG, posterior inferior frontal gyrus; Put, putamen; SN, substantia nigra; SFG, superior frontal gyrus; SPL, superior parietal lobule; STG, superior temporal gyrus; STS, superior temporal sulcus; TP, temporal pole; vmPFC, ventromedial prefrontal cortex; VTA, ventral tegmental area.
